# A Micro-Machined Gyroscope for Rotating Aircraft

**DOI:** 10.3390/s120709823

**Published:** 2012-07-23

**Authors:** Qingwen Yan, Fuxue Zhang, Wei Zhang

**Affiliations:** 1 School of Electronic Engineering, Beijing University of Posts and Telecommunications, Beijing 100876, China; 2 Sensing Technique Research Center, Beijing Information Science and Technology University, Beijing 100101, China; E-Mails: zhangfuxue@263.net (F.Z.); way_zh@163.com (W.Z.)

**Keywords:** silicon micromachining, gyroscope, rotating aircraft

## Abstract

In this paper we present recent work on the design, fabrication by silicon micromachining, and packaging of a new gyroscope for stabilizing the autopilot of rotating aircraft. It operates based on oscillation of the silicon pendulum between two torsion girders for detecting the Coriolis force. The oscillation of the pendulum is initiated by the rolling and deflecting motion of the rotating carrier. Therefore, the frequency and amplitude of the oscillation are proportional to the rolling frequency and deflecting angular rate of the rotating carrier, and are measured by the sensing electrodes. A modulated pulse with constant amplitude and unequal width is obtained by a linearizing process of the gyroscope output signal and used to control the deflection of the rotating aircraft. Experimental results show that the gyroscope has a resolution of 0.008 °/s and a bias of 56.18 °/h.

## 1. Introduction

Improvement of control and stability systems of aircrafts largely depends on improving the accuracy and reliability of instruments for measuring their spatial movement. Meanwhile, since the airborne equipment is seriously crowded in the cabin, attention is paid to the miniaturization of mechanical and electrical instruments, which include gyroscopes and stability systems. Therefore a new gyroscope should be small in size, lightweight with low power consumption and low price. In the 1990s, a wide variety of silicon micro-machined gyroscopes appeared with the development of microelectronics technology, but most of the silicon micro-machined gyroscopes used static electrical force to drive the mass and sense the angular rate of the carrier in the sense mode [1,2]. In general the performance of vibrating rate gyroscope is strongly influenced by two characteristic modes of motion: (i) the first or fundamental mode defining the drive frequency; and (ii) the secondary mode defining the sense motion [3,4]. Compared with older gyroscopes, a new gyroscope driven by a rotating carrier is presented, which only has sense motion mode [5,6]. The drive force comes from the roll of the carrier, so the signal of gyroscope contains the information of the roll rate of the carrier.

## 2. Design and Fabrication

The silicon pendulum of the micro-machined gyroscope contains many damping slots suspended by two torsion girders that are anchored to the outer frame. By applying the roll rate, *φ̇*, to the outer frame, the silicon pendulum rotates with the outer frame about the Z axis. An angular rate, *Ω*, applied about the X axis, generates a Coriolis force that acts to push the pendulum in and out of the frame of oscillation, *i.e.*, sense motion.

In general sense motion is a forced damped vibration and can be described by a rotational angle *α*, torque *K_T_*, the moment of inertia *J_x_*, *J_y_*, *J_z_* and damping factor *δ*. The roll rate and deflection rate of the carrier are *φ̇* and *Ω*:

(1) $$J_{Y}\ddot{\alpha }+\delta \dot{\alpha }+\left[\left(J_{Z}-J_{X}\right){\dot{\varphi }}^{2}+K_{T}\right]\alpha =\left(J_{Z}+J_{Y}-J_{X}\right)\Omega \dot{\varphi }\cos\left(\dot{\varphi }t\right)$$

(2) $$\alpha =Ae^{-(\delta /2J_{Y})t}\cos\left(\sqrt{\frac{1}{J_{Y}}[(J_{Z}-J_{X}){\dot{\varphi }}^{2}+K_{T}]-\frac{\delta^{2}}{4J_{Y}}}t+\chi \right)+B\cos(\dot{\varphi }t)$$

(3) $$B=\frac{\left(J_{Z}+J_{Y}-J_{X}\right)/\dot{\varphi }\Omega }{\sqrt{{\left[\left(J_{Z}-J_{Y}-J_{X}\right){\dot{\varphi }}^{2}+K_{T}\right]}^{2}+{\left(\delta \dot{\varphi }\right)}^{2}}}$$

Equation (2) describes the whole solution of the Equation (1). The first part of Equation (2) attenuates quickly, with the transient amplitude *A*, its phase shift *χ*. The second part of Equation (2) depends on the Coriolis force, with the amplitude *B*. The stationary solution of Equation (1) is:

(4) $$\alpha =\frac{\left(J_{Z}+J_{Y}-J_{X}\right)\dot{\varphi }\Omega \cos\left(\dot{\varphi }t\right)}{\sqrt{{\left[\left(J_{Z}-J_{Y}-J_{X}\right){\dot{\varphi }}^{2}+K_{T}\right]}^{2}+{\left(\delta \dot{\varphi }\right)}^{2}}}$$

**Figure 1.**
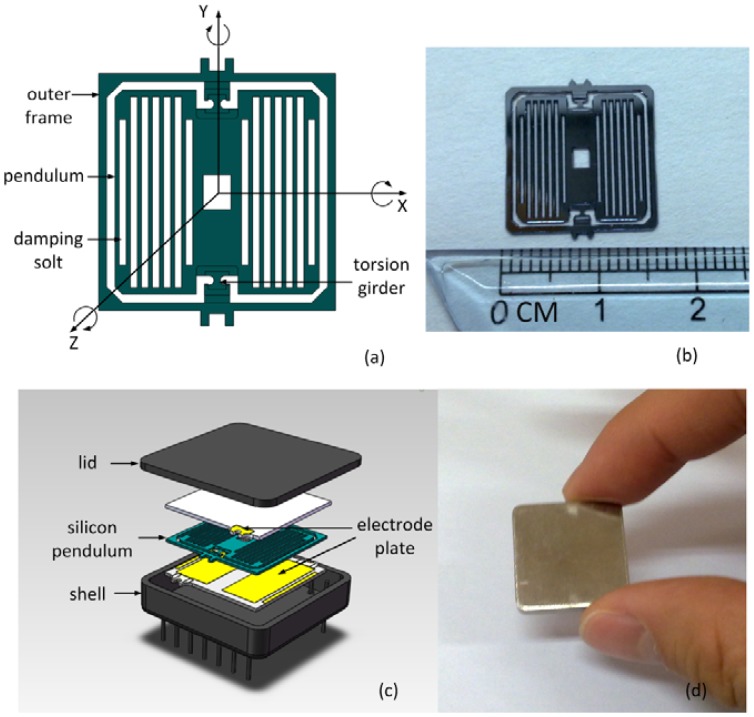
(**a**) Structure of silicon pendulum; (**b**) Silicon pendulum picture; (**c**) Expanded solid model showing the silicon pendulum, electrode plate, lid and shell; (**d**) Gyroscope picture.

Figure 1(a) shows the sensor structure. The fundamental frequency of the gyroscope was calculated at 490 Hz by finite element analysis. Starting from a standard 4″ two-sided polished silicon wafer, the first thick thermal oxide layers are grown. In the first lithography, silicon oxide etching and silicon etching step, the pendulum thickness and the outer frame are made. The second thick thermal oxide layers are grown. Onto this pendulum, the damping slots are opened in the second lithography, silicon oxide etching and silicon etching step. The pendulum is released in the third and fourth lithography, silicon oxide etching and silicon etching step. The third thick thermal oxide layers are grown. The torsion girders are released in the fifth lithography, silicon oxide etching and silicon etching step. A picture of the silicon pendulum is shown in Figure 1(b). Two electrode plates are glued on the silicon chip encapsulating the whole pendulum element. The shell and lid provide a hermetical sealing, shown in Figure 1(c,d).

## 3. Application

A special application of the gyroscope involves using it in the autopilot of a rotating aircraft. The gyroscope signal is the amplitude modulation signal. The change in signal amplitude reflects the change of input angular rate, and the change in signal frequency reflects the change of the roll rate of the rotating aircraft. Therefore, the autopilot of rotating aircraft can directly utilize the gyroscope signal without any hybrid frequency signal. The gyroscope signal, *U_t_*, adds the linearized signal, *U_s_*, to linearize the gyroscope signal, so the amplitudes of gyroscope signal change into the time of across zero. The gyroscope signal is:

(5) $$U_{t}\left(t\right)=K\Omega \cos\left(\dot{\varphi }t\right)$$

where *K* is the gyroscope scale factor.

The linearized signal is:

(6) $$U_{s}\left(t\right)=U_{sm}\cos\left(\omega_{s}t\right)$$

where *U_sm_* is the amplitude of linearized signal, and *ω_s_* is the frequency of linearized signal. The combination signal *U_k_*(*t*) is:

(7) $$U_{k}\left(t\right)=U_{t}\left(t\right)+U_{s}\left(t\right)=K\Omega \cos\left(\dot{\varphi }t\right)+U_{sm}\cos\left(\omega_{s}t\right)$$

**Figure 2.**
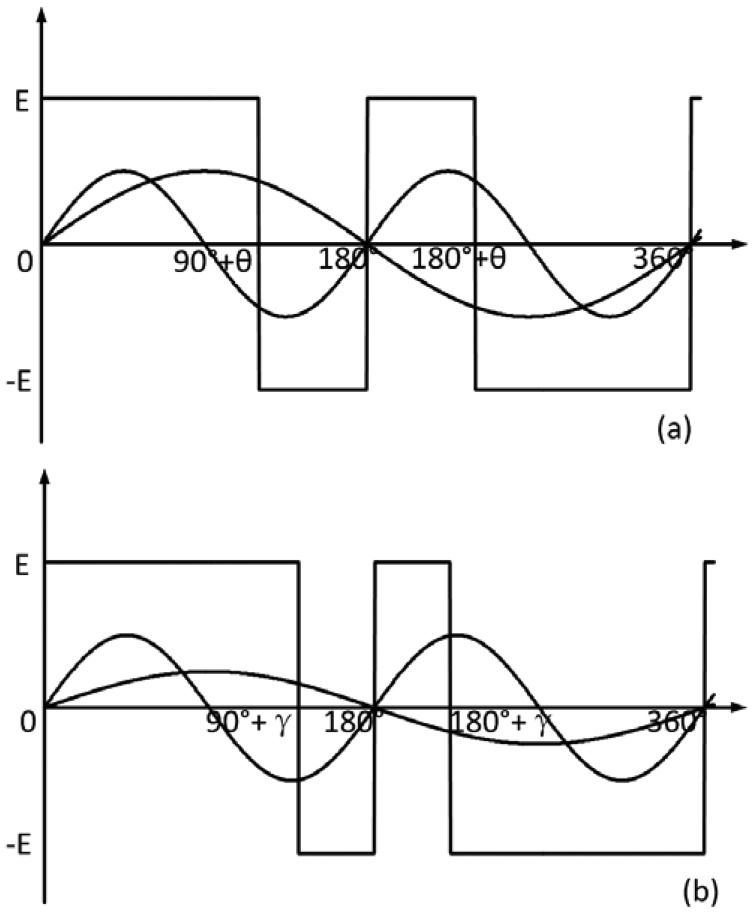
(**a**) Waveform of the gyroscope signal, the linearized signal and the control signal in the first assumption; (**b**) Waveform of the gyroscope signal, the linearized signal and the control signal in the second assumption.

The control signal of the autopilot of a rotating aircraft *U_dk_*(*t*) is a modulated pulse signal. *U_dk_*(*t*) is positive when *U_k_*(*t*) > 0, and *U_dk_*(*t*) is negative when *U_k_*(*t*) < 0. For analysis convenience, some assumptions will be made. The first assumption is that the amplitude and frequency of the linearized signal are twice those of the gyroscope signal. The control signal switches polarity at 90° + θ, 180°, 180° + θ, 360° in one roll circle, as shown in Figure 2(a). The second assumption is that the amplitude of the linearized signal is equal to that of the gyroscope signal, and the frequency of the linearized signal is twice that of the gyroscope signal. The control signal switches polarity at 90° + γ, 180°, 180° + γ, 360° in one roll circle, as shown in Figure 2(b). The θ and γ are the phases of the control signal. Therefore the modulated pulse is the signal with a constant amplitude and unequal time width. The rudders of the rotating aircraft change the direction at the time when the pulse crosses zero, so they change about four times in one rolling circle of the rotating aircraft [7,8].

## 4. Results

Two tests were performed on a three-axis turntable to verify that the gyroscope signal satisfies Equation (2), *i.e.*, the frequency of gyroscope signal is equal to the roll rate of the rotating carrier, and the amplitude of gyroscope is proportional to the deflect angular rate of the rotating carrier.

**Figure 3.**
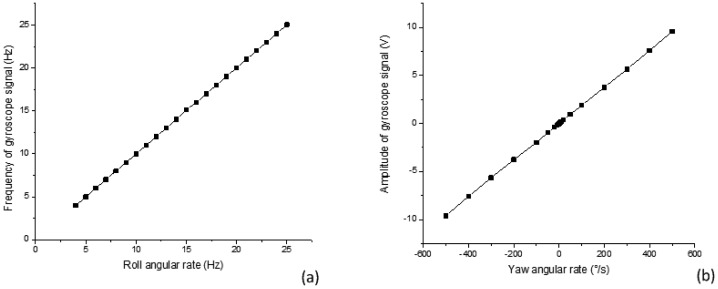
(**a**) Frequency of gyroscope signal as a function of roll angular rate; (**b**) Amplitude of gyroscope signal as a function of yaw angular rate.

In the first test the yaw angular rate is set at 20 °/s, the roll angular rate changes from 4 Hz to 25 Hz. The results of this experiment are shown in Figure 3(a).

In the second test the roll angular rate is set at 17 Hz, the yaw angular rate changes from negative 500 °/s to positive 500 °/s. The results of this experiment are shown in Figure 3(b). The test data shows good results and strong promise for the gyroscope performance. The main performance specifications for the gyroscope are summarized in Table 1.

## 5. Conclusions

A new gyroscope was designed to meet the requirements for application in the autopilot of a rotating aircraft. The gyroscope uses the roll of the rotating aircraft as a driver, so the frequency of the gyroscope signal is equal to the roll rate of the rotating aircraft. Moreover, the roll of the rotating aircraft can make the gyroscope sense the Coriolis force in 360° space, so the gyroscope signal is linearized and can always be used to control the rudder. The outstanding performance proves the conceptual approach is feasible for application in the field.

## Figures and Tables

**Table 1.** The main performance specifications of the gyroscope.

**No**	**Parameters**	**Unit**	**No: 0801-4003**

01	Input angular rate limit	°/s	±500
02	Scale factor	mv/(°/s)	15.7
03	Linearity error of scale factor	%	0.5
04	Asymmetry of scale factor	%	0.039
05	Repeatability of scale factor	ppm	14.36
06	Resolution	°/s	0.008
07	Temperature sensitivity of scale factor	ppm/°C	1,086.01
08	Bias	°/h	56.18
09	Repeatability of bias	°/h	65.59
10	Noise floor	°/h/√Hz	13.72
